# Effect of Different Selenium Species on Indole-3-Acetic Acid Activity of Selenium Nanoparticles Producing Strain *Bacillus altitudinis* LH18

**DOI:** 10.3390/molecules29112463

**Published:** 2024-05-23

**Authors:** Mengjun Li, Rui Yang, Nana Li, Siyang Zhao, Shiya Wei, Sishang Zhang, Jue Gong, Jie Song, Jun-Ran Kim, Yi He, Chao Gao, Zhangqian Wang, Shuiyuan Cheng

**Affiliations:** 1 National R&D Center for Se-Rich Agricultural Products Processing, School of Modern Industry for Selenium Science and Engineering, Wuhan Polytechnic University, Wuhan 430023, Chinaheyi629@126.com (Y.H.);; 2 Hubei National Se-Rich Technology Development Co., Ltd., Enshi 445000, China; 3 Suixian Hongfa Native Co., Ltd., Suizhou 431500, China; 4 Plant Quarantine Technology Research and Development, Animal and Plant Quarantine Agency, Gimcheon-si 39660, Gyeongsangbuk-do, Republic of Korea

**Keywords:** *Bacillus altitudinis*, sodium selenite, selenium nanoparticles (SeNPs), indole-3-acetic acid (IAA)

## Abstract

Acting as a growth regulator, Indole-3-acetic acid (IAA) is an important phytohormone that can be produced by several *Bacillus* species. However, few studies have been published on the comprehensive evaluation of the strains for practical applications and the effects of selenium species on their IAA-producing ability. The present study showed the selenite reduction strain *Bacillus altitudinis* LH18, which is capable of producing selenium nanoparticles (SeNPs) at a high yield in a cost-effective manner. Bio-SeNPs were systematically characterized by using DLS, zeta potential, SEM, and FTIR. The results showed that these bio-SeNPs were small in particle size, homogeneously dispersed, and highly stable. Significantly, the IAA-producing ability of strain was differently affected under different selenium species. The addition of SeNPs and sodium selenite resulted in IAA contents of 221.7 µg/mL and 91.01 µg/mL, respectively, which were 3.23 and 1.33 times higher than that of the control. This study is the first to examine the influence of various selenium species on the IAA-producing capacity of *Bacillus* spp., providing a theoretical foundation for the enhancement of the IAA-production potential of microorganisms.

## 1. Introduction

Selenium is one of the trace elements required by animals, plants, and humans [1], which has a wide range of biological effects, including anti-oxidation [2], antitumor [3], and enhancing human immunity [4,5]. At low doses, selenium can enhance growth, photosynthesis, and nutrient homeostasis in plants [6,7]. Natural oxidation states of selenium include elemental selenium(0), selenide(-II), selenite(IV), and selenate(VI) [8,9]. Red selenium nanoparticles (SeNPs) in elemental selenium have a variety of biological functions and a wide range of applications. Studies have shown that the toxicity of SeNPs is lower than that of selenite [10] and that SeNPs have higher bioavailability and greater potential for use in various biomedical and environmental applications. Many strains have been reported to biosynthesize SeNPs, including *Streptomyces* spp. [11], *Azospirillum brasilens* [12], *Lactobacillus* spp. [13,14], *Bacillus* spp. [15,16,17], *Stenotrophomonas maltophilia* [18], and *Pseudomonas alcaliphila* [19], which are capable of synthesizing uniformly sized spherical SeNPs both inside and outside the cell. More importantly, in recent years, biological treatment involving microorganisms has been considered the preferred and sustainable alternative due to its low cost and ecofriendliness. The particle size, stability [20], antioxidant [21], antimicrobial [22], and anticancer [23] properties of SeNPs have been extensively investigated, but there are fewer comprehensive evaluations of their growth-promoting ability and environmental adaptability as fertilizers or nutrient solutions.

Furthermore, it has been shown that soil microorganisms can play an important role in biogeochemistry [24], especially in the secretion of active substances, such as indoleacetic acid, from soil or crops. Studies have shown that inoculation of exogenous IAA-producing bacteria can enhance the activity of antioxidant enzymes in plants [25]. By producing IAA, these bacteria can alter the auxin pool in the plant to an optimal or superoptimal level, leading to improved plant growth and development [26]. Therefore, it is of great significance to use IAA-producing bacteria to produce bioorganic fertilizer. The use of microorganisms as an environmentally friendly strategy to support crop production has enormous potential. It has been shown that *Bacillus circulans* E9 has different ability to produce IAA in LB and PYM media, 3.73 ± 0.15 µg/mL and 7.81 ± 0.16 µg/mL, respectively [27]. The study reported an IAA yield of 48.75 ± 3 µg/mL after 72 h of incubation of *K. pneumoniae,* and the treatment was optimized by the response surface methodology (RSM) tool [28]. However, little research has been carried out to investigate the effect of adding selenium to the culture medium on its ability to produce IAA and even less on the addition of different selenium species.

This study aimed to isolate a strain with high selenium tolerance and identify it as *B. altitudinis* LH18 by colony morphology observation, physiological and biochemical measurements, and molecular biology techniques. The biogenic SeNPs produced by the strain were systematically characterized by DLS, zeta potential analysis, SEM, and FTIR. Moreover, to evaluate the environmental adaptability of the strain LH18 in question, its tolerance to acid, alkali, salt, and the heavy metal cations Cd(II) was determined. To further explore the integrated application ability of strain LH18, the effects of adding different selenium species on the ability of strain LH18 to produce IAA were determined. It greatly enriches the selenium-enriched microbial resources and broadens the application of selenium-enriched microorganisms and SeNPs in the plant field.

## 2. Results

### 2.1. Isolation and Identification of Strains

#### 2.1.1. Isolation and Screening

The higher the concentration of sodium selenite, the lighter the red color of the strain, indicating that the strain has limited tolerance to selenium (Figure S1A). In this study, selenite-reducing *Bacillus* selenite was isolated from soil from LB plates spiked with 300 mmol/L sodium selenite (Figure S1B). Among all the strains, the isolated strain LH18 was in a better growth condition and could reduce sodium selenite to red SeNPs. After 72 h of incubation at 37 °C and 180 rpm, it was observed that strain LH18 could reduce a maximum of 250 mmol/L sodium selenite to red SeNPs (Figure S1C). Therefore, this strain was selected for further studies.

**Figure 1:**
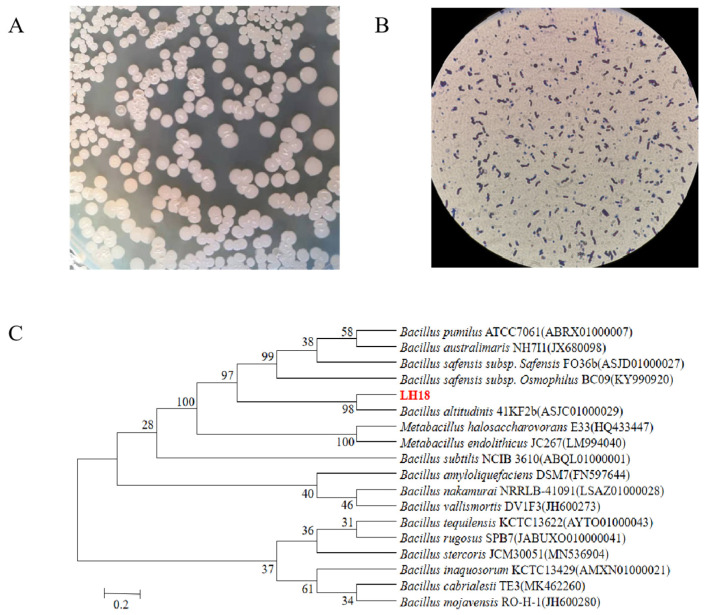
Characterization of strain LH18. (**A**) Identification of colony morphology. (**B**) Gram staining of strain LH18. (**C**) Neighbor-joining tree inferred through MEGA 6 software based on the sequences of 16S rDNA gene, showing the phylogenetic relationship of strain LH18 and related species obtained from the Ezbiocloud website.

Bacterial strains were identified by extracting the bacterial DNA followed by DNA amplification using universal primers 27F/1492R. The extracted DNA was sent to Shanghai Shenggong sequencing company for sequencing, and the obtained sequences were submitted to GenBank for accession numbers. The 16S rDNA had a length of 1454 bp, and its sequence was most similar to *Bacillus altitudinis* LH18. Phylogenetic analysis based on the neighbor of joining method also shows isolated LH18 clustered with *Bacillus altitudinis* (Figure 1). More importantly, the results of physiological and biochemical tests (Table S1) showed that the isolate LH18 possessed biochemical and physiological character *B. altitudinis*. Based on the results of the above analysis, it can be concluded that the bacterium is *B. altitudinis* LH18.

#### 2.1.2. Characterization of Selenium-Enriching Bacteria

**Figure 2:**
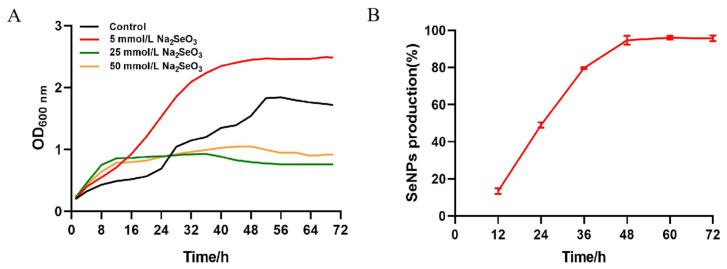
Growth curve, selenite reduction in *B. altitudinis* LH18 in the presence of selenite (5 mmol/L). (**A**) Growth curve of strain LH18 in the different concentrations of selenite (0, 5, 25, and 50 mmol/L). (**B**) Selenite reduction in *B. altitudinis* LH18 in the presence of selenite (5 mmol/L).

The bacterial growth curve was evaluated using four different concentrations of selenite, viz. 0, 5, 25, and 50 mmol/L in LB (Figure 2); the reduction process was observed to start concomitantly with microbial growth without any significant lag phase. However, it will have a certain inhibitory effect on the growth of the strain when the concentration arrives at 25 mmol/L. Moreover, in the medium containing sodium selenite, the culture medium first became turbid, and the color gradually changed to red when it reached the logarithmic phase (between 12 and 24 h of incubation).

Similarly, it showed that sodium selenite reduction was correlated closely to growth kinetics in *Rhodopseudomonas palustris* strain N [29]. The reduction occurred between the end of the exponential phase and the beginning of the stationary phase. According to the description, 79% of sodium selenite exhaustion happened during the exponential development phase of *B. fungorum* DBT1, and the maximum amount of decrease happened during the stationary growth phase of *B. fungorum* 95 [30]. Furthermore, as the concentration of sodium selenite provided by the same bacterium varies, so does the primary reduction period. In *Bacillus mycoides* SeITE01 and in the presence of 0.5 mmol/L sodium selenite, the total amount of sodium selenite initially added to the cultures was exhausted during the exponential phase of growth. By contrast, when 2.0 mmol/L sodium selenite was supplied, the selenite was mainly depleted during the stationary phase.

In cultures of the *B. altitudinis* LH18 strain with an initial sodium selenite concentration of 5 mmol/L, depletion of sodium selenite occurs during the exponential growth phase (between 6 and 24 h of incubation). The results show that 49% of the initial sodium selenite was depleted within 24 h, of which only 13% occurred within the first 12 h (Figure 2). At the end of the full 48 h logarithmic period, the conversion rate was 94.69%. The largest reduction occurred between 24 and 48 h. The 6 h delay before the onset of reduction can be explained by the slow sodium selenite uptake during the initial growth described elsewhere.

### 2.2. Determination of the Tolerance Capacity of Strain LH18

#### 2.2.1. Determination of Acid and Alkali Tolerance by Strain LH18

**Figure 3:**
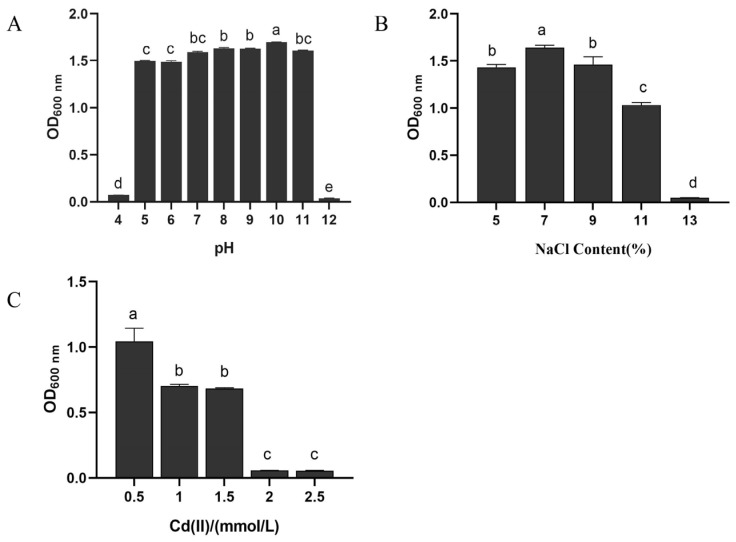
Effects of different pH (**A**), NaCl concentrations (**B**), and Cd(II) (**C**) on the growth of *B. altitudinis* LH18. The different alphabetic superscripts in the same column are significantly different (*p* < 0.05) based on Tukey’s multiple comparison test.

The effect of different pH on the growth of *B. altitudinis* LH18 (Figure 3A) significantly affected the growth, reproduction, and physiological metabolism of microorganisms. In the process of fermentation, the influence of pH is mainly reflected in the growth and reproduction of microorganisms, metabolic synthesis, and the physical and chemical properties of fermentation broth. According to the experimental results, *B. altitudinis* LH18 could grow in pH 5–11, indicating that *B. altitudinis* LH18 could grow in extreme environments and has a wide range of environmental adaptability.

#### 2.2.2. Determination of Salt Tolerance by Strain LH18

The optimum growth salt concentration of weak halophilic microorganisms is 0.2–0.5 mol/L, the optimum growth salt concentration of moderate halophilic microorganisms is 0.5–2.5 mol/L, and the optimum growth salt concentration of extreme halophilic microorganisms is 2.5–5.2 mol/L. After 72 h of culture, *B. altitudinis* LH18 could grow in 11% NaCl (1.88 mol/L), indicating that *B. altitudinis* LH18 is a moderately halophilic bacterium that can grow at high salt concentrations (Figure 3B).

#### 2.2.3. Determination of Heavy Metal Cations Resistance Cd(II)

The following experiment was carried out to determine whether strain LH18 can grow in an environment with high levels of Cd(II). *B. altitudinis* LH18 was able to grow in 1.5 mmol/L Cd(II) solution, indicating that the strain has the ability to tolerate the heavy metal cation Cd(II) (Figure 3C). Compared with previous research, the ability of this strain to tolerate Cd(II) is at a better level among the *Bacillus* species and provides a microbial resource for heavy metal degradation.

### 2.3. Characterization of SeNPs

#### 2.3.1. Dynamic Light Scattering (DLS) Analysis

**Figure 4:**
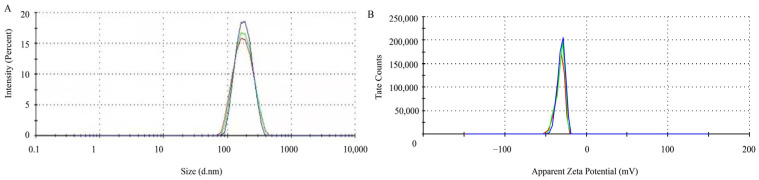
The characteristics of SeNPs produced by *B. altitudinis* LH18. (**A**) Particle size distribution. (**B**) zeta potential.

The particle size and size distribution of the SeNPs were analyzed by DLS. The results of DLS analysis indicated that the size distribution of purified SeNPs was 228.5 nm (Figure 4), and the SeNPs have a high zeta potential. The results showed that the zeta potential of SeNPs was −31.5 mV, indicating that the SeNPs produced by *B. altitudinis* LH18 were stable.

#### 2.3.2. Scanning Electron Microscopy (SEM) Analysis

**Figure 5:**
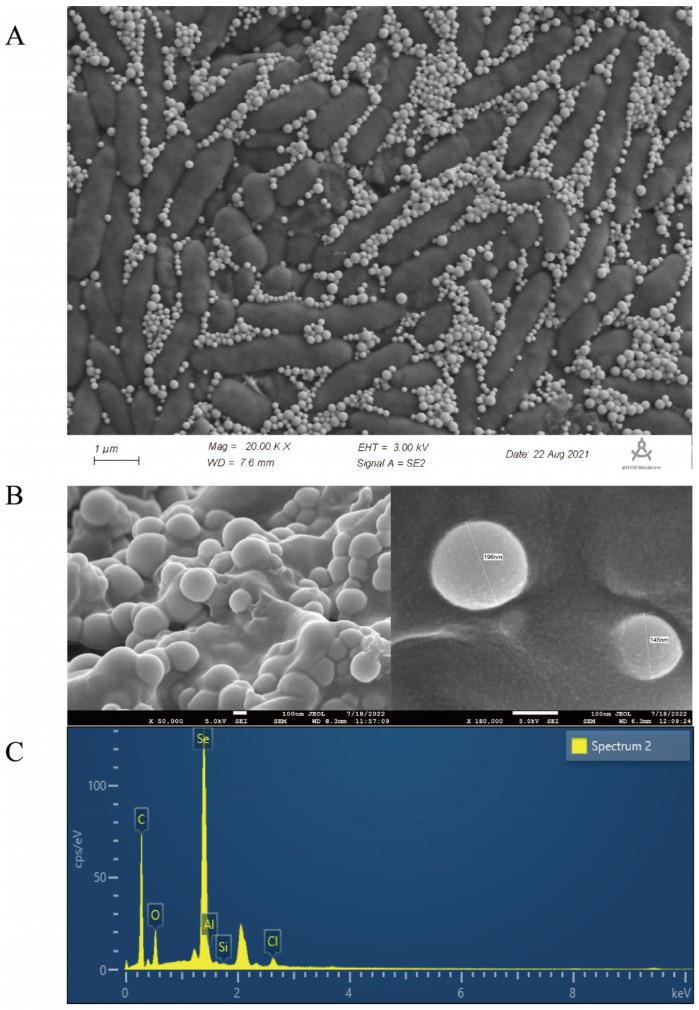
SEM and EDS of *B. altitudinis* LH18 and SeNPs grown with sodium selenite. (**A**) SEM image of *B. altitudinis* LH18 and SeNPs. (**B**) SEM image of Purified SeNPs. (**C**) EDS spectrum of Purified SeNPs.

To elucidate the effect of sodium selenite reduction and SeNP production on the morphology of strain LH18, it was inoculated into a medium containing 5 mmol/L sodium selenite. SEM analysis showed the morphology of *B. altitudinis* LH18 and SeNPs after 48 h incubation in a sodium selenite-containing medium (Figure 5). Scanning electron microscopy results showed that SeNPs coexisted on the surface of *B. altitudinis* LH18 (Figure 5A), *B. altitudinis* LH18 was rod-shaped with a size of about 2 µm, and SeNPs were spherical with a uniform size distribution and a small particle size of about 228.5 nm (Figure 5B). The extraction yielded purified SeNPs, which were spherical in shape and rounded by a layer of material on the surface. Studies [31] showed that SeNPs are surrounded by a layer of sugar or protein, so it is speculated that the material may be sugar or protein. Energy-dispersive X-ray (EDX) analysis showed that the purified SeNPs were spherical and clearly indicated the presence of selenium (Figure 5C). The size of SeNPs is an important factor in determining chemical properties and biological activity. These results indicate that *B. altitudinis* LH18 can produce small-sized and highly stable SeNPs, which constitutes a multifunctional platform that may be suitable for biotechnology applications.

#### 2.3.3. Fourier Transform Infrared Spectrometer (FTIR) Analysis

**Figure 6:**
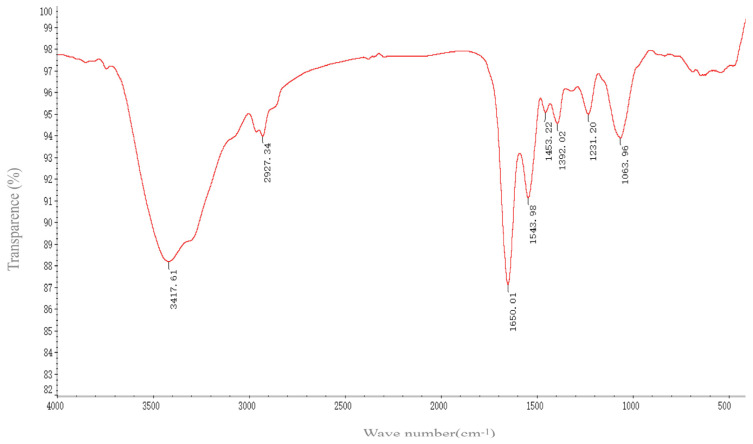
FTIR micrographs of SeNPs produced by *B. altitudinis* LH18.

FTIR spectroscopy revealed the functional groups on the surface of SeNPs by measuring the vibration frequency in the chemical functional groups. The groups such as N-H, O-H, CH_2_, CH_3_, C=O, and C-O-C existing on the surface of the biogenic SeNPs produced by *B. altitudinis* LH18 may be coated by proteins and polysaccharides that were involved in the formation and stabilization of the SeNPs (Figure 6). The various absorption bands and corresponding distributions in the FTIR spectrum were as follows: The FTIR spectrum of SeNPs shows an absorption band at 3417.61 cm^−1^, corresponding to the overlap of N-H and O-H stretching vibrations of the amino group and hydroxyl group of the carboxyl group in the amino acid [32]. The peak centered at 2927.34 cm^−1^ (broad and asymmetric) was a sum of the various stretching vibrations of the C-H moieties in the methyl and methylene groups, largely in protein side chains, while the peak at 1650.01 cm^−1^ indicated the presence of C=O (amide I band) in the amide group [33]. The peak at 1543 cm^−1^ corresponded to the N-H plane bending of protein amide II. The weak and broad peak centered at 1453.22 cm^−1^ evidently contains contributions from various vibrations, such as the stretching of C-N, the bending of C-H, and the symmetric stretching modes of ionized carboxylic groups. The peak at 1392.02 cm^−1^ could be attributed to C=O of COO– symmetric stretching in proteins. The broad peak at 1063.96 cm^−1^ was located in the typical C-O/C-C/C-N vibration modes characteristic of polysaccharides and/or proteins. In consequence, FTIR spectroscopy confirmed the presence of proteins and possible polysaccharides on the surface of the resulting biological SeNPs (amide I: 1650.01 cm^−1^, amide II: 1543.98 cm^−1^, and amide III: 1231.2 cm ^–1^) and carbohydrates (1063.96 cm^−1^). This indicates that the extracellular polymers secreted by *B. altitudinis* LH18 are coated on the surface of SeNPs, and the presence of these extracellular polymers is beneficial to the long-term stable storage of SeNPs.

### 2.4. Production of IAA Capacity

#### 2.4.1. Effect of Adding Different Concentrations of Sodium Selenite on IAA Production

**Figure 7:**
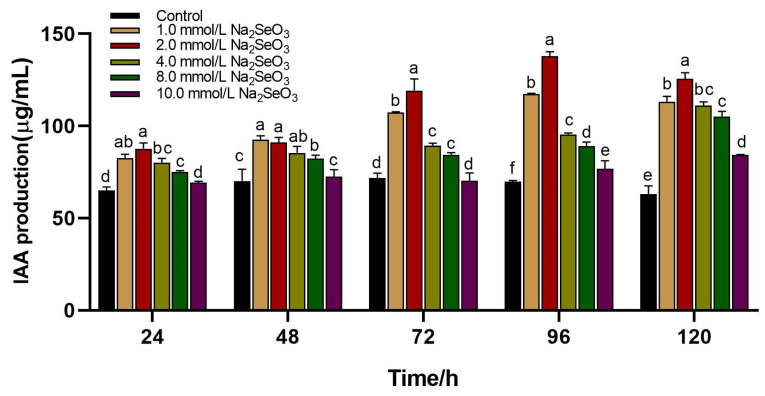
Effect of different concentrations of sodium selenite on IAA production capacity of *B. altitudinis* LH18; mean values with the different letters are significant (*p* ≤ 0.05).

IAA is a plant growth hormone that can directly or indirectly regulate plant growth and development. IAA is typically produced naturally by plants and can also be obtained from soil and microbial communities. The synthesis of IAA can proceed through two distinct routes. The non-tryptophan pathway includes the method by which IAA can be made without the inclusion of L-tryptophan and its derivatives. Second, the tryptophan pathway is involved in the production of IAA by the addition of L-tryptophan. The results showed that after adding 2 mmol/L of sodium selenite, the solution color was redder, and IAA production capacity was better after incubation at 37 °C for 4 d (Figure 7). The results showed that the yield of IAA was 137.98 µg/mL, 1.99 times that of the control. Therefore, 2 mmol/L was selected as the additional selenium source in the later period.

#### 2.4.2. Effects of Different Selenium Species on IAA Production

**Figure 8:**
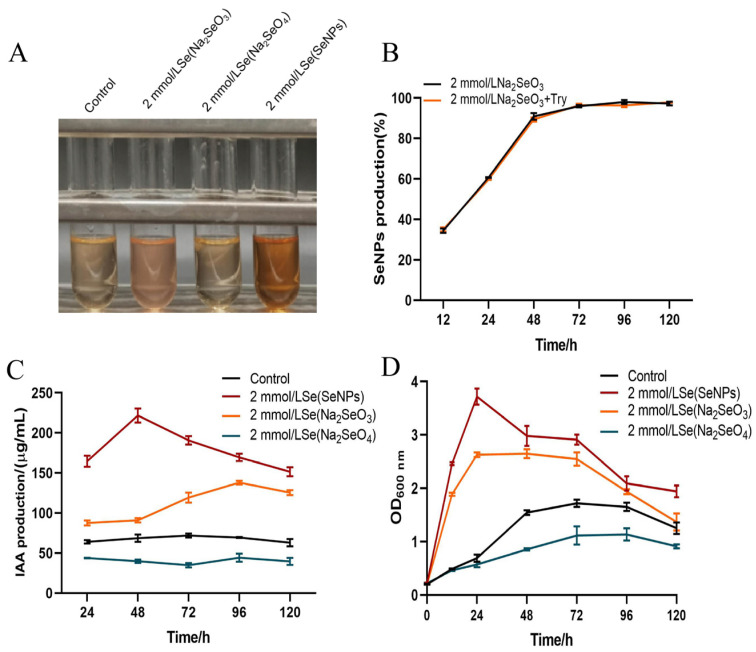
Effect of the addition of different selenium species on the IAA-producing activity of *B. altitudinis* LH18 and the ratio of sodium selenite reduction to SeNPs. (**A**) Color of IAA-producing activity of strain LH18. (**B**) Effect of different selenium species on IAA production ability of *B. altitudinis* LH18. (**C**) Effect of different selenium species on IAA production ability of *B. altitudinis* LH18. (**D**) Effects of different selenium species on the growth status of *B. altitudinis* LH18.

The results of the study observed the effect of adding different selenium species to the culture medium on the ability of strain LH18 to produce IAA. After two days of incubation, the treatment with the addition of SeNPs significantly enhanced its ability to produce IAA, followed by the sodium selenite and sodium selenate groups (Figure 8A). The addition of tryptophan did not significantly affect the yield of strain LH18 on the production of SeNPs using sodium selenite (Figure 8B). From the results, it was known that 2 mmol/L treatments of SeNPs and sodium selenite increased the ability of the strain to produce IAA, and sodium selenite treatment decreased the ability of the strain to produce IAA. The amount results (Figure 8C) showed that the ability of IAA production was improved after adding 2 mmol/L SeNPs and sodium selenite at 37 °C for 48 h. IAA production was 221.7 µg/mL and 91.01 µg/mL, respectively, which were 3.23 times and 1.33 times higher than that of the control group. After 96 h culture, the IAA content of the SeNP group and sodium selenite group were 169.46 µg/mL and 137.98 µg/mL, respectively, which were 2.44 times and 1.99 times that of the control group. However, after adding 2 mmol/L sodium selenate, the IAA production ability of *B. altitudinis* LH18 decreased.

To explore the mechanism, we also evaluated the growth status of the strain. The results showed that the addition of SeNPs resulted in the largest growth of the strain and was directly proportional to the strain’s IAA-producing ability, followed by the control, sodium selenite, and sodium selenate in descending order of the growth of strain LH18. After the addition of sodium selenate, the growth of strain LH18 was reduced compared with the control group. The reason may be that sodium selenate has a destructive effect on the cell walls of microorganisms, so some microorganisms cannot survive and die normally, resulting in a decrease in the ability of microorganisms to produce IAA.

## 3. Discussion

In addition, studies have shown that selenium can also increase the germination rate of seeds, promote plant growth, and increase chlorophyll content in leaves. Selenium has been demonstrated in numerous research to promote plant growth; however, fewer studies have looked at how Se affects microorganisms’ capacity to release IAA. In this experiment, a strain of selenium-tolerant *B. altitudinis* LH18 was screened, the highest selenium-tolerant concentration of the strain was determined in plate culture and liquid, and the growth kinetic curve of the strain was determined. This paper showed that the maximum growth of strain LH18 was significantly increased by the addition of 5 mmol/L sodium selenite. The absorbance values measured in the mid-late logarithmic and stabilization phases of the culture were higher than the maximum values obtained in the absence of sodium selenite. The same phenomenon has been observed in the research [34]. After analyzing several potential explanations, it was determined that one of them might be the way selenium encouraged the strains’ growth, which raised their biomass.

This study demonstrated that *B. altitudinis* can reduce 94.69% sodium selenite to SeNPs in 48 h. Moreover, the size of SeNPs is closely related to their inactivity, and usually, the smaller the particle size, the more bioactive they are. Studies have shown that the wavelength range of most biogenic SeNPs is 100–500 nm. The biological SeNPs produced by this strain had good stability, as evidenced by the results, which revealed that at pH 7, they were spherical, tiny, and uniformly dispersed, with an average particle size of 228.5 nm, a PDI of 0.412, and a zeta potential of −31.5 mV.

In this study, it was found that *B. altitudinis* LH18 could grow in 1.5 mmol/L Cd(II) solution. In comparison to previous studies [35,36], the microbial strain showed superior resistance to the heavy metal cation Cd(II), and it was demonstrated that the strain is also capable of producing IAA. IAA is a plant auxin that is released by bacteria that support plant growth. It not only encourages seed germination but also controls plant growth. IAA can promote plant growth by regulating plant cell division and related gene expression, and it plays an important role in agricultural production [37]. Studies have shown that applying selenium compounds as fertilizers can boost plant yields. For instance, adding sodium selenite, organic selenium, selenocysteine, and SeNPs can boost plant production [38]. Moreover, applying selenium to cadmium-contaminated soil protects and stimulates plants, which can help strains become more acclimated to their surroundings [39].

At present, the research of microbial strains capable of producing SeNPs mainly focuses on antioxidation, antivirus, and improving immunity. However, there are few studies on the IAA production ability of the strain and the production and activity of IAA by different selenium species. To evaluate the potential of the strain as a growth-promoting bacteria, the ability of LH18 to produce indole acetic acid was also explored, and the results showed that strain LH18 has a strong ability to secrete indole acetic acid. Under the same growth conditions, the addition of different selenium species had different effects on the ability of strain LH18 to produce IAA.

After 48 h incubation, the IAA content of the SeNP group and sodium selenite group were 221.7 µg/mL and 91.01 µg/mL, respectively, which were 3.23 times and 1.33 times higher than that of the control group. After 96 h culture, the IAA content of the SeNP group and sodium selenite group were 169.46 µg/mL and 137.98 µg/mL, respectively, which were 2.44 times and 1.99 times of the control group. Through analysis, it is possible that the addition of SeNPs can increase the number of viable bacteria, thus promoting the IAA production capacity of *B. altitudinis* LH18. To create selenium-enriched microorganisms and products that have the capacity to manufacture IAA, this offers theoretical direction as well as technological assistance. To the best of our knowledge, there is no published literature on the effect of different selenium species on the capacity of *Bacillus* spp. to produce IAA. Furthermore, this investigation examined the impact of several selenium species on the capacity of strains to generate IAA in order to improve the microbial resources enriched with selenium.

## 4. Materials and Methods

### 4.1. Screening and Isolation of Selenium-Tolerant Bacterial Strains

The soil samples were selected from soil layers near the selenium mine in Enshi Autonomous Prefecture, Hubei Province. Briefly, 1 g of soil sample was diluted serially (10-fold) in sterilized distilled water and heated at 65 °C for 1 h. Then, 100 µL of each dilution was spread on LB plates and cultured at 37 °C for 24 h. Cultures were isolated and purified by continuous streaking to obtain single colonies. A single colony was picked to place on LB plates containing 200 mmol/L and 300 mmol/L sodium selenite and cultured at 37 °C for 24 h. Because colonies with red color indicate those with selenite reduction and selenium(0) formation, individual colonies with red color were picked and subcultured on new LB plates. A total of 22 strains resistant to 300 mmol/L selenite were screened out. The seed solution of strains was inoculated with 2% inoculation amount into LB liquid medium containing 0, 50, 100, 150, 200, 250, and 300 mmol/L selenite, respectively, and incubated for 72 h in a constant temperature shaker at 37 °C at 180 rpm to observe and record the growth of the cell and the production of SeNPs.

### 4.2. Identification of Selenium-Tolerant Bacterial Strains

To identify and isolate LH18, biochemical and physiological analyses were conducted using standard methods. The colonial morphology and biochemical of the strain were observed, as described in the “Bergey’s Manual of Systematic Bacteriology”. The single colony was analyzed using the standard Gram staining method. 16S rDNA genes were obtained by PCR using the isolated genomic DNAs as templates and a pair of universal primers, 27F and 1492R. Then, the 16S rDNA gene sequence was compared with those available on the EzBioCloud server [40]. A Neighbor of Joining tree was constructed using MEGA 7.0 software, and bootstrap confidence values were obtained using 1000 resamplings.

### 4.3. Characterization of Selenium-Enriching Bacteria

#### 4.3.1. Dynamics of Microbial Growth

To assess the effect of selenite on bacterial cells, the growth curves of LH18 were determined in the presence of 0, 5, 25, and 50 mmol/L selenite in LB broth. Screened Se-enriched bacteria were inoculated into 100 mL of LB medium (approximately OD_600_ = 0.03) in 250 mL conical flasks at 37 °C. Therefore, we measured the cell concentration (OD_600_) once every 2 h throughout the culture and plotted the growth curve.

#### 4.3.2. Determination of SeNPs Production

Using inductively coupled plasma mass spectrometry (ICP-MS) to measure the amount of sodium selenite that remains in the medium can help assess the sodium selenite bioreducing rate [41]. The samples underwent centrifugation at a force of 12,000× *g* for a duration of 20 min. Thus, the resulting supernatant was carefully transferred to a fresh tube and subjected to filtration using a 0.22 µm filter, enabling the analysis of the remaining sodium selenite concentration within the supernatant. The filtered supernatant was digested in 3 mL of concentrated HNO_3_ overnight and passed through a 0.22 µm filter. Subsequently, the samples were diluted to the appropriate selenium concentration and analyzed by ICP-MS.

### 4.4. Determination of the Tolerance Capacity of Strain LH18

#### 4.4.1. Determination of the Adaptability of Strain LH18 to Acidic and Alkaline Environments

The single colony of *B. altitudinis* LH18 was selected and cultured in an LB medium overnight for 12 h. The OD_600_ was measured to be about 0.8. The LB solution of different pH (4–12) was inoculated at 2% inoculation amount and cultured at 37 °C and 180 rpm. The OD_600_ was measured at 72 h.

#### 4.4.2. Determination of Salt Tolerance by Strain LH18

LB media containing 5, 7, 9, 11, and 13% NaCl were prepared, respectively. The single colony was picked and activated by an LB medium for 12 h, and the bacterial solution was diluted with an LB medium to OD_600_ to 0.8. The above LB liquid medium containing different concentrations of NaCl was inoculated at an inoculum size of 2%, a total volume of 100 mL, 37 °C, 180 rpm. Samples were taken at 72 h, respectively, and the absorbance of the bacteria was measured at 600.

#### 4.4.3. Determination of the Tolerance Capacity of Strain LH18 to the Heavy Metal Cations Cd(II)

The single colony of *B. altitudinis* LH18 was selected and cultured in an LB medium overnight for 12 h (approximately OD_600_ = 0.8). According to the 2% inoculation amount, it was inoculated into different concentrations of Cd(II) (0.5, 1, 1.5, 2, and 2.5%) LB solution, 37 °C, 180 rpm culture, sampling 72 h to measure OD_600_.

### 4.5. Preparation and Characterization of SeNPs

#### 4.5.1. Separation and Purification of SeNPs

The seed liquid of the activated strain (OD_600_ = 0.8) was inoculated into LB liquid medium containing a certain concentration of sodium selenite at 180 rpm by 1% inoculation amount, and the fermentation broth was cultured at 37 °C until the fermentation broth became red. The fermentation broth containing SeNPs was collected in a centrifuge tube, placed in a centrifuge at 12,000 rpm, centrifuged for 10 min, and the precipitate was collected as a mixture of bacteria and SeNPs. The sediment should be cleaned with sterile water two or three times. Then, it should be put in a mortar and liquid nitrogen added right away for grinding. After grinding, the bacterial powder was suspended with sterile water, and the cells on the ice were ultrasonically broken for 10 min. The SeNP suspension was filtered through 10, 5, 3, 1.8, 1.2, 1, 0.8, and 0.45 μm membranes, respectively. The SeNP suspension was moved to the separator funnel, and one-fifth volume of n-Hexane was added. The mixture was fully mixed and stood, and the lower layer was collected as the product. After centrifugation at 12,000 rpm for 10 min, the precipitate was collected and washed with sterile water 2–3 times. The precipitate was taken and freeze-dried to obtain SeNPs.

#### 4.5.2. Dynamic Light Scattering (DLS) Analysis

The degree of dispersion and stability of SeNPs can be ascertained by DLS by measuring the average size and zeta potential of the particles. This technique works on the principle of light scattering. The light was scattered by the SeNPs, and thus, the average size of SeNPs was obtained with its polydispersity index (PDI) [42]. A DLS instrument was used to distribute the mean size of SeNPs. The refractive index was 1.33, and the absorption coefficient was 1.0 [43].

#### 4.5.3. Scanning Electron Microscopy (SEM) Analysis

Strain LH18 was grown in LB supplemented with 5 mmol/L sodium selenite at 37 °C, 180 rpm. After 12 h of cultivation, cells were centrifuged (8000 rpm, 10 min). Harvested centrifuged were washed thrice with phosphate-buffered saline (PBS, pH 8.0). Fixation was conducted with 2.5% glutaraldehyde (24 h, 4 °C). Finally, freeze dried and sputter coated. The samples were then viewed using SEM (JSM-6390 JEOL, Tokyo, Japan).

#### 4.5.4. Fourier Transform Infrared Spectrometer (FTIR) Analysis

To identify the major structural groups surrounded by the purified SeNPs, FTIR analysis was performed. Firstly, the freeze-dried KBr was ground into powder and then placed into an infrared dryer for drying. Then, 1 mg SeNPs was mixed evenly with KBr at a ratio of 1:100, loaded into a configured die, and pressed at 20 MPa. The film thickness obtained after pressing is 0.4 mm, and the pressed film is analyzed. FTIR analysis was performed to find out the function present on the SeNPs. This equipment involves the use of infrared radiation (IR) to obtain the peaks of the samples.

### 4.6. Determination of IAA Production Capacity of Strain LH18

#### 4.6.1. Potential for IAA Production Capacity of Strain LH18

To measure the ability of *B. altitudinis* LH18 to produce IAA, single colonies were inoculated in an LB medium containing 100 mg/L L-tryptophan and cultured at 37 °C and 180 rpm for 1 d. Then, the bacterial solution was centrifugated for 8000 rpm. After 10 min, 50 µL of supernatant was put on the white ceramic plate. The same volume of Salkowski colorimetric agent (50 mL 35% HClO_4_ and 1 mL 0.5 mg/mL FeCl_3_) was added and mixed well. Moreover, 50 mL of 20 mg/L IAA standard solution was added to the third column of the white ceramic plate as a positive control, and the color change was observed after 30 min of reaction in the dark at room temperature.

#### 4.6.2. Determination of IAA Production Capacity

IAA standard solutions with concentrations of 0, 10, 5, 20, 30, 40, and 50 mg/L were mixed with Salkowski colorimetric reagent (1:1) and reacted in the dark for 30 min, and the absorbance was measured at 530 nm using a UV spectrophotometer. An IAA standard curve was drawn with IAA concentration as the abscissa and absorbance value as the ordinate. Strain LH18 was inoculated into an LB medium and cultured for 5 d at 37 °C and 180 rpm. The filtered culture medium was mixed with a Salkowski colorimetric reagent (1:1) and reacted in the dark for 30 min. To obtain the IAA yield of the strain, the absorbance of the mixture was measured at 530 nm, and this value was substituted into the IAA standard curve.

#### 4.6.3. Effect of Sodium Selenite on the Production of IAA by Strain LH18

In an LB medium supplemented with L-tryptophan, 0, 1, 2, 4, 8, and 10 mmol/L sodium selenite were added, respectively. The content of IAA was measured after incubation at 37 °C and 180 rpm for 4 d. At the same time, the OD_600_ of different groups was measured by UV, and the rate of reducing sodium selenite was measured by the sodium sulfide method.

#### 4.6.4. Effects of Different Selenium Species on IAA Production

Different selenium species (sodium selenate, sodium selenite, and SeNPs) of 2 mmol/L were added to an LB medium supplemented with L-tryptophan (100 mg/L) content of IAA was measured after incubation at 37 °C and 180 rpm for 2 d. At the same time, the OD_600_ of different groups was measured by UV, and the rate of reducing sodium selenite was measured by the sodium sulfide method.

## 5. Conclusions

In this study, a bacterial strain with extreme tolerance to sodium selenite was isolated from the soil layer near the selenium mine, with a tolerance of up to 300 mmol/L, indicating that more SeNPs might be produced. The smaller particle size of SeNPs synthesized by strain LH18 indicates that it has better biological activity. The prepared SeNPs were characterized by DLS, zeta potential, SEM, and FTIR. The results showed that the SeNPs were spherical, with small and uniformly dispersed particles and good stability. In addition, the comprehensive application ability of the strain was evaluated. The strain has good resistance to acid, alkali, salt, and heavy metal cations Cd(II) and has extensive biological adaptability. In addition, the effects of three different selenium species on the IAA production capacity of *B. altitudinis* LH18 were discussed. The results showed that the addition of sodium selenite and SeNPs could significantly improve the IAA production capacity of *B. altitudinis* LH18. This is the first time study of the effect of different selenium species on IAA production by *Bacillus* spp. The discovery of this strain enriched microbial resources and provided theoretical support for improving the ability of microorganisms to produce IAA.

## Data Availability

The data are available on request.

## Supplementary Materials

The following supporting information can be downloaded at: https://www.mdpi.com/article/10.3390/molecules29112463/s1, Figure S1. Screening of selenium-tolerant strains. Growth of the strain on 200 mmol/L (A) and 300 mmol/L (B) sodium selenite plates. (C) Tolerance of strain LH18 to different concentrations of sodium selenite. Table S1. Differential characteristics of strain LH18 and *Bacillus altitudinis*.

## References

[1] Chauhan R., Awasthi S., Srivastava S., Dwivedi S., Pilon-Smits E.A.H., Dhankher O.P., Tripathi R.D. (2019). Understanding selenium metabolism in plants and its role as a beneficial element. Crit. Rev. Environ. Sci. Technol..

[2] Dai Z.H., Ding S., Chen J.Y., Han R., Cao Y., Liu X., Tu S., Guan D.X., Ma L.Q. (2022). Selenate increased plant growth and arsenic uptake in As-hyperaccumulator *Pteris vittata* via glutathione-enhanced arsenic reduction and translocation. J. Hazard. Mater..

[3] Liu S., Hu J., Li M., Zhu S., Guo S., Guo H., Wang T., Zhang Y., Zhang J., Wang J. (2021). The role of Se content in improving anti-tumor activities and its potential mechanism for selenized *Artemisia sphaerocephala* polysaccharides. Food Funct..

[4] Pezzarossa B., Remorini D., Gentile M.L., Massai R. (2012). Effects of foliar and fruit addition of sodium selenate on selenium accumulation and fruit quality. J. Sci. Food Agric..

[5] Kushwaha A., Goswami L., Lee J., Sonne C., Brown R.J.C., Kim K.-H. (2021). Selenium in soil-microbe-plant systems: Sources, distribution, toxicity, tolerance, and detoxification. Crit. Rev. Environ. Sci. Technol..

[6] Chauhan R., Awasthi S., Tripathi P., Mishra S., Dwivedi S., Niranjan A., Mallick S., Tripathi P., Pande V., Tripathi R.D. (2017). Selenite modulates the level of phenolics and nutrient element to alleviate the toxicity of arsenite in rice (*Oryza sativa* L.). Ecotoxicol. Environ. Saf..

[7] Rizwan M., Ali S., Zia-Ur-Rehman M., Rinklebe J., Ok Y.S. (2020). Effects of selenium on the uptake of toxic trace elements by crop plants: A review. Crit. Rev. Environ. Sci. Technol..

[8] Lenz M., Lens P.N. (2009). The essential toxin: The changing perception of selenium in environmental sciences. Sci. Total Environ..

[9] Nakamaru Y., Tagami K., Uchida S. (2005). Distribution coefficient of selenium in Japanese agricultural soils. Chemosphere.

[10] Ullah A., Yin X., Wang F., Xu B., Mirani Z.A., Xu B., Chan M.W.H., Ali A., Usman M., Ali N. (2021). Biosynthesis of Selenium Nanoparticles (via *Bacillus subtilis* BSN313), and Their Isolation, Characterization, and Bioactivities. Molecules.

[11] Tan Y., Yao R., Wang R., Wang D., Wang G., Zheng S. (2016). Reduction of selenite to Se(0) nanoparticles by filamentous bacterium *Streptomyces* sp. ES2-5 isolated from a selenium mining soil. Microb. Cell Factories.

[12] Tugarova A.V., Vetchinkina E.P., Loshchinina E.A., Burov A.M., Nikitina V.E., Kamnev A.A. (2014). Reduction of selenite by *Azospirillum brasilense* with the formation of selenium nanoparticles. Microb. Ecol..

[13] Li Z., Wang Q., Dai F., Li H. (2022). Reduction of selenite to selenium nanospheres by Se(IV)-resistant *Lactobacillus paralimentarius* JZ07. Food Chem..

[14] Palomo-Siguero M., Gutiérrez A.M., Pérez-Conde C., Madrid Y. (2016). Effect of selenite and selenium nanoparticles on lactic bacteria: A multi-analytical study. Microchem. J. Devoted Appl. Microtech. All Branches Sci..

[15] Lampis S., Zonaro E., Bertolini C., Bernardi P., Butler C.S., Vallini G. (2014). Delayed formation of zero-valent selenium nanoparticles by *Bacillus mycoides* SeITE01 as a consequence of selenite reduction under aerobic conditions. Microb. Cell Factories.

[16] Duan Y., Li M., Zhang S., Wang Y., Deng J., Wang Q., Yi T., Dong X., Cheng S., He Y. (2022). Highly Efficient Biotransformation and Production of Selenium Nanoparticles and Polysaccharides Using Potential Probiotic *Bacillus subtilis* T5. Metabolites.

[17] Wang Y., Yu Y., Duan Y., Wang Q., Cong X., He Y., Gao C., Hafeez M., Jan S., Rasheed S.M. (2022). Enhancing the Activity of Carboxymethyl Cellulase Enzyme Using Highly Stable Selenium Nanoparticles Biosynthesized by *Bacillus paralicheniformis* Y4. Molecules.

[18] Lampis S., Zonaro E., Bertolini C., Cecconi D., Monti F., Micaroni M., Turner R.J., Butler C.S., Vallini G. (2017). Selenite biotransformation and detoxification by *Stenotrophomonas maltophilia* SeITE02: Novel clues on the route to bacterial biogenesis of selenium nanoparticles. J. Hazard. Mater..

[19] Kora A.J., Rastogi L. (2016). Biomimetic synthesis of selenium nanoparticles by Pseudomonas aeruginosa ATCC 27853: An approach for conversion of selenite. J. Environ. Manag..

[20] Blinov A.V., Nagdalian A.A., Siddiqui S.A., Maglakelidze D.G., Gvozdenko A.A., Blinova A.A., Yasnaya M.A., Golik A.B., Rebezov M.B., Jafari S.M. (2022). Synthesis and characterization of selenium nanoparticles stabilized with cocamidopropyl betaine. Sci. Rep..

[21] Xu C., Qiao L., Guo Y., Ma L., Cheng Y. (2018). Preparation, characteristics and antioxidant activity of polysaccharides and proteins-capped selenium nanoparticles synthesized by *Lactobacillus casei* ATCC 393. Carbohydr. Polym..

[22] Serov D.A., Khabatova V.V., Vodeneev V., Li R., Gudkov S.V. (2023). A Review of the Antibacterial, Fungicidal and Antiviral Properties of Selenium Nanoparticles. Materials.

[23] Ullah A., Mu J., Wang F., Chan M.W.H., Yin X., Liao Y., Mirani Z.A., Sebt E.H.S., Aslam S., Naveed M. (2022). Biogenic Selenium Nanoparticles and Their Anticancer Effects Pertaining to Probiotic Bacteria-A Review. Antioxidants.

[24] Sokol N.W., Slessarev E., Marschmann G.L., Nicolas A., Blazewicz S.J., Brodie E.L., Firestone M.K., Foley M.M., Hestrin R., Hungate B.A. (2022). Life and death in the soil microbiome: How ecological processes influence biogeochemistry. Nat. Rev. Microbiol..

[25] Gamalero E., Glick B.R. (2022). Recent Advances in Bacterial Amelioration of Plant Drought and Salt Stress. Biology.

[26] Iqbal M., Wagi S., Ahmed A. (2018). Open Access *Phyllospheric* Bacterial Treatments Improve Growth in *Helianthus annuus* L. RADS J. Biol. Res. Appl. Sci..

[27] Sarmiento-López L.G., López-Meyer M., Maldonado-Mendoza I.E., Quiroz-Figueroa F.R., Sepúlveda-Jiménez G., Rodríguez-Monroy M. (2022). Production of indole-3-acetic acid by *Bacillus circulans* E9 in a low-cost medium in a bioreactor. J. Biosci. Bioeng..

[28] Biswas S., Philip I., Jayaram S., Sarojini S. (2023). Endophytic bacteria *Klebsiella* spp. and *Bacillus* spp. from *Alternanthera philoxeroides* in Madiwala Lake exhibit additive plant growth-promoting and biocontrol activities. J. Genet. Eng. Biotechnol..

[29] Li B., Liu N., Li Y., Jing W., Fan J., Li D., Zhang L., Zhang X., Zhang Z., Wang L. (2014). Reduction of selenite to red elemental selenium by *Rhodopseudomonas palustris* strain N. PLoS ONE.

[30] Khoei N.S., Lampis S., Zonaro E., Yrjala K., Bernardi P., Vallini G. (2017). Insights into selenite reduction and biogenesis of elemental selenium nanoparticles by two environmental isolates of *Burkholderia fungorum*. New Biotechnol..

[31] Dat N.M., Huong L.M., Cong C.Q., Hai N.D., Nam N.T.H., Thinh D.B., Duy H.K., Danh T.T., Loi P., Phong M.T. (2022). Green synthesis of chitosan-based membrane modified with uniformly micro-sizing selenium particles decorated graphene oxide for antibacterial application. Int. J. Biol. Macromol..

[32] Chen J., Chen X., Li J., Luo B., Fan T., Li R., Liu X., Song B., Jia X., Zhong S. (2022). Preparation and Characterization of Nano-Selenium Decorated by Chondroitin Sulfate Derived from Shark Cartilage and Investigation on Its Antioxidant Activity. Mar. Drugs.

[33] Kamnev A.A., Dyatlova Y.A., Kenzhegulov O.A., Vladimirova A.A., Mamchenkova P.V., Tugarova A.V. (2021). Fourier Transform Infrared (FTIR) Spectroscopic Analyses of Microbiological Samples and Biogenic Selenium Nanoparticles of Microbial Origin: Sample Preparation Effects. Molecules.

[34] Avendaño R., Chaves N., Fuentes P., Sánchez E., Jiménez J.I., Chavarría M. (2016). Production of selenium nanoparticles in *Pseudomonas putida* KT2440. Sci. Rep..

[35] Rahman Z., Singh V.P. (2019). The relative impact of toxic heavy metals (THMs) (arsenic (As), cadmium (Cd), chromium (Cr)(VI), mercury (Hg), and lead (Pb)) on the total environment: An overview. Environ. Monit. Assess..

[36] Matsuda R., Handayani M.L., Sasaki H., Takechi K., Takano H., Takio S. (2018). Production of indoleacetic acid by strains of the epiphytic bacteria *Neptunomonas* spp. isolated from the red alga *Pyropia yezoensis* and the seagrass *Zostera marina*. Arch. Microbiol..

[37] Ahmed A., Hasnain S. (2008). Auxin producing *Bacillus* sp.: Auxin quantification and effect on the growth of *Solanum tuberosum*. J. Biotechnol..

[38] Viltres P.M., Sánchez M.M.J., Llugany M., Boada R., Valiente M. (2024). Selenium biofortification of microgreens: Influence on phytochemicals, pigments and nutrients. Plant Physiol. Biochem..

[39] Seregina I.I., Trukhachev V.I., Belopukhov S.L., Dmitrevskaya I.I., Zhevnerov A.V., Zharkikh O.A. (2023). The use of selenium for protective and stimulating effects on plants when soil is contaminated with cadmium. Braz. J. Biol..

[40] Yoon S.H., Ha S.M., Kwon S., Lim J., Kim Y., Seo H., Chun J. (2017). Introducing EzBioCloud: A taxonomically united database of 16S rRNA gene sequences and whole-genome assemblies. Int. J. Syst. Evol. Microbiol..

[41] Baggio G., Groves R.A., Chignola R., Piacenza E., Presentato A., Lewis I.A., Lampis S., Vallini G., Turner R.J. (2021). Untargeted Metabolomics Investigation on Selenite Reduction to Elemental Selenium by *Bacillus mycoides* SeITE01. Front. Microbiol..

[42] Sanie Z., Iqbal M.S., Abbas K., Qadir M.I. (2022). Synthesis, characterization and evaluation of biological properties of selenium nanoparticles from *Solanum lycopersicum*. Arab. J. Chem..

[43] Zhu Y., Ren B., Li H., Lin Z., Bañuelos G., Li L., Zhao G., Guo Y. (2018). Biosynthesis of selenium nanoparticles and effects of selenite, selenate, and selenomethionine on cell growth and morphology in *Rahnella aquatilis* HX_2_. Appl. Microbiol. Biotechnol..

