# Supplementary material for: Effect of Different Selenium Species on Indole-3-Acetic Acid Activity of Selenium Nanoparticles Producing Strain Bacillus altitudinis LH18
Source: Molecules. 2024 May 23;29(11):2463. doi: 10.3390/molecules29112463 (PMC11173968; doi:10.3390/molecules29112463)
Supplement: Supplementary file 1 [file molecules-29-02463-s001.zip › molecules-2968261-supplementary.pdf]

A

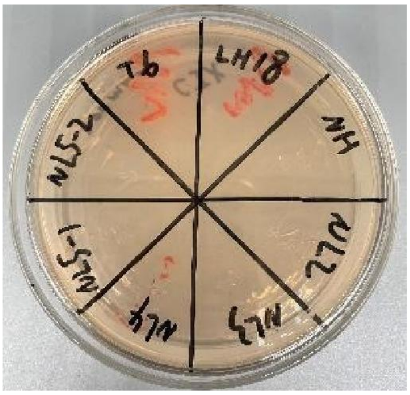

B

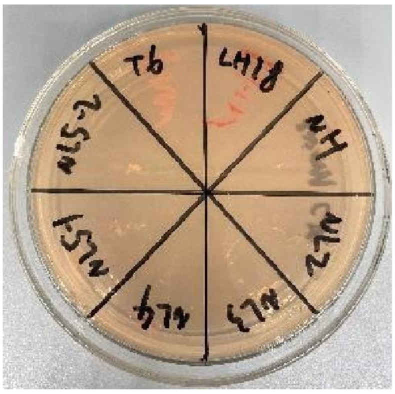

Control 50 mmol/L 100 mmol/L 150 mmol/L 200 mmol/L 250 mmol/L 300 mmol/L

C

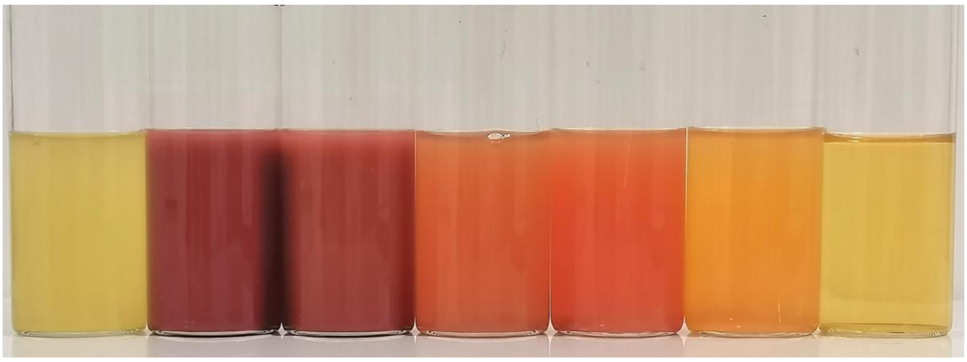

Figure S1. Screening of selenium-tolerant strains. Growth of the strain on 200 mmol/L (A) and 300 mmol/L (B) sodium selenite plates. (C) Tolerance of strain LH18 to different concentrations of sodium selenite.

Table S1. Differential characteristics of strain LH18 and *Bacillus altitudinis*.

**Characteristic**	**Strain**	
	**Strain LH18**	***Bacillus altitudinis***
Glucose	+	+
Sucrose	+	+
Lactose	-	-
Mannose	+	+
Arabinose	+	+
Xylose	+	+
V-P experiment	+	+

[1] +Positive -Negative
